# A *MAPKKK* gene from rice, *RBG1res*, confers resistance to *Burkholderia glumae* through negative regulation of ABA

**DOI:** 10.1038/s41598-023-30471-9

**Published:** 2023-03-09

**Authors:** Ritsuko Mizobuchi, Kazuhiko Sugimoto, Seiya Tsushima, Shuichi Fukuoka, Chikako Tsuiki, Masaki Endo, Masafumi Mikami, Hiroaki Saika, Hiroyuki Sato

**Affiliations:** 1 Institute of Crop Science, National Agriculture and Food Research Organization (NARO), 2-1-2 Kannondai, Tsukuba, Ibaraki 305-8518 Japan; 2 Strategic Planning Headquarters, NARO, 3-1-1 Kannondai, Tsukuba, Ibaraki 305-8517 Japan; 3 Institute of Agrobiological Sciences, NARO, 3-1-3 Kannondai, Tsukuba, Ibaraki 305-8604 Japan; 4 Present Address: Core Technology Research Headquarters, NARO, 3-1-1 Kannondai, Tsukuba, Ibaraki 305-8517 Japan

**Keywords:** Genetics, Plant sciences

## Abstract

*Burkholderia glumae* causes bacterial seedling rot (BSR) of rice and is a threat to a consistent food supply. When previously screening for resistance against *B. glumae* in the resistant cultivar Nona Bokra (NB) versus the susceptible cultivar Koshihikari (KO), we detected a gene, *Resistance to Burkholderia glumae 1* (*RBG1*), at a quantitative trait locus (QTL). Here, we found that *RBG1* encodes a *MAPKKK* gene whose product phosphorylates OsMKK3. We also found that the kinase encoded by the *RBG1* resistant (*RBG1res*) allele in NB presented higher activity than did that encoded by the *RBG1* susceptible (*RBG1sus*) allele in KO. *RBG1res* and *RBG1sus* differ by three single-nucleotide polymorphisms (SNPs), and the G390T substitution is essential for kinase activity. Abscisic acid (ABA) treatment of inoculated seedlings of RBG1res-NIL (a near-isogenic line (NIL) expressing *RBG1res* in the KO genetic background) decreased BSR resistance, indicating that *RBG1res* conferred resistance to *B. glumae* through negative regulation of ABA. The results of further inoculation assays showed that RBG1res-NIL was also resistant to *Burkholderia plantarii*. Our findings suggest that *RBG1res* contributes to resistance to these bacterial pathogens at the seed germination stage via a unique mechanism.

## Introduction

*Burkholderia glumae* is a seedborne plant pathogen that causes bacterial seedling rot (BSR) and bacterial grain rot (BGR) of rice^1^, both of which are serious diseases around the world. This disease is referred to as bacterial panicle blight. Instances of BSR and BGR are predicted to increase under global warming because the optimal growth temperature range for the pathogen is relatively high (30–35 °C)^2,3^. Since *B. glumae* was first found in 1955 in Japan^4–7^, it has been reported in other regions, such as the USA^8,9^, East and South Asia^10–17^, Latin America^18,19^, and South Africa^20^. According to a recent report^21^, under a scenario in which the daily temperature increases by 1 °C, production losses caused by *B. glumae* would cause an economic loss equivalent to US$112 million per year in the USA and a decrease in production equivalent to that required to feed 2.17 million people. Although seed treatment with oxolinic acid, a quinoline derivative, has been used for bacterial disease control in Japan^22–24^, many strains of *B. glumae* tolerant to oxolinic acid have been found^25,26^. Therefore, it is necessary to discover quantitative trait loci (QTLs) that confer resistance to *B. glumae* to enable breeding of resistant cultivars. By comparing the BSR severity in cultivars from the World Rice Collection (WRC) and other sources, we found widespread diversity among resistance, and Nona Bokra (NB), which is a traditional lowland indica cultivar that originated in India, was found to have the highest resistance to BSR^27^.

To identify candidate genes for resistance to BSR, we selected two cultivars exhibiting contrasting phenotypes to the pathogen in a preliminary screening: NB, which showed the highest resistance, and Koshihikari (KO), which is a susceptible modern lowland rice cultivar^27^. In a subsequent step, from among 44 chromosome segment substitution lines (CSSLs) generated from a cross between NB and KO^28^, we previously found that SL535 was resistant^29^. We mapped the BSR resistance of SL535 to chromosome 10 and designated the region as the *quantitative trait locus for RESISTANCE TO BACTERIAL SEEDLING ROT 1* (*qRBS1*). Because the gene at this QTL is the first identified QTL for resistance to *B. glumae*, its name was renamed *Resistance to Burkholderia glumae 1* (*RBG1*)^30^.

Here, we report that *RBG1* encodes a *MAPKKK* gene that negatively regulates abscisic acid (ABA) and that RBG1 is an upstream kinase of OsMKK3*.* A near-isogenic line (NIL) (RBG1res-NIL) containing *RBG1res* in the KO genetic background showed resistance not only to *B. glumae* but also to bacterial seedling blight caused by *Burkholderia plantarii.* Our results suggest that *RBG1res* is a promising candidate for the genetic improvement of resistance against *Burkholderia* strains that negatively affect rice germination.

## Results

### Phenotypic characteristics and map-based cloning of *RBG1res*

**Figure 1:**
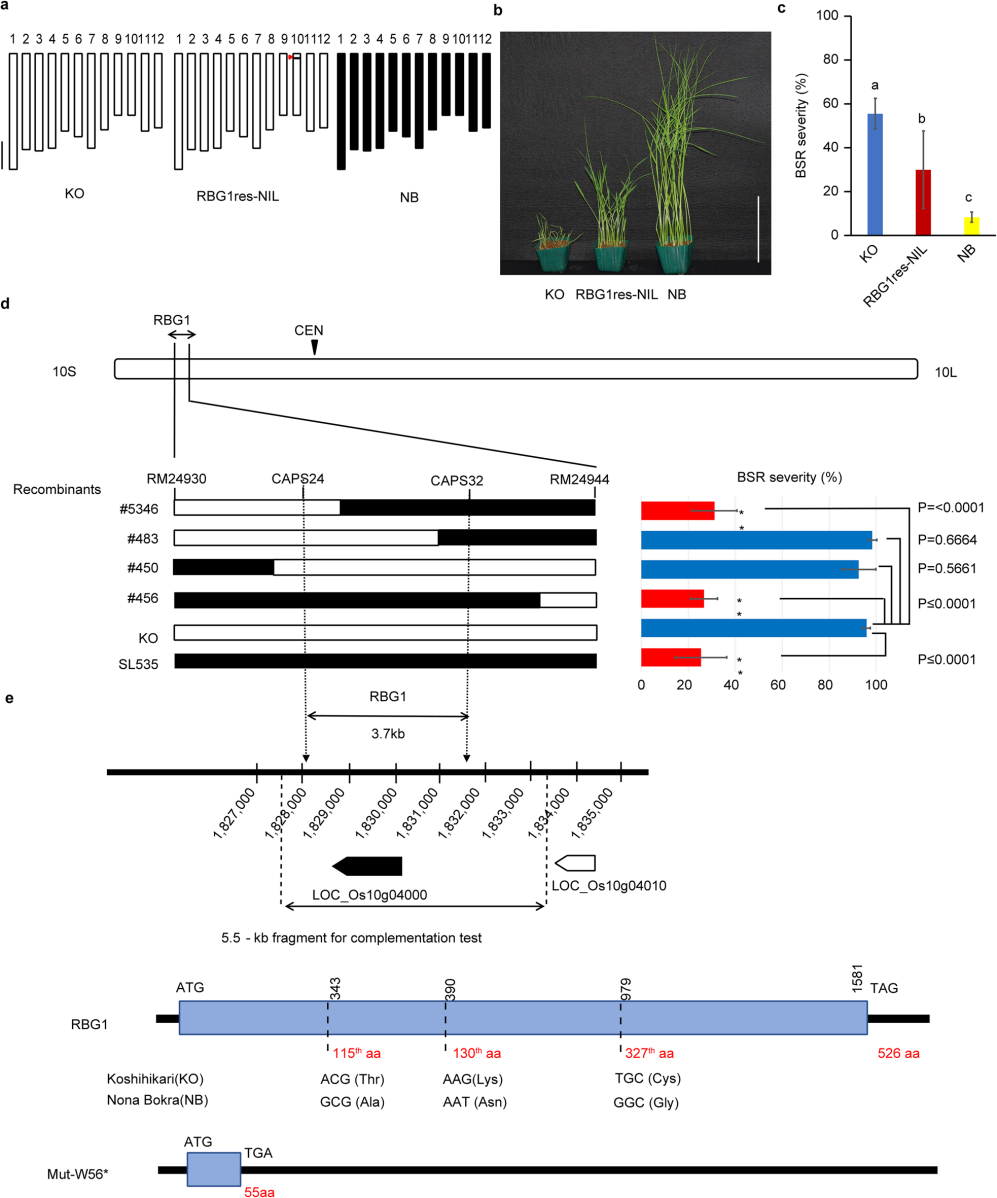
Phenotypic and molecular characterization of *RBG1res.* (**a**) Graphical genotypes (chromosomes 1–12) of KO (left), RBG1res-NIL (centre), and NB (right). The white and black rectangles indicate homozygous regions from KO and NB, respectively. The red arrowhead shows the position of *RBG1res*. The scale bar represents 10 Mb. (**b**) Differences in resistance to BSR among KO, RBG1res-NIL, and NB. The image shows symptoms of 7-day-old seedlings after inoculation with *B. glumae* at 10^8^ CFU/ml. The scale bar represents 10 cm. (**c**) BSR severity of inoculated KO, RBG1res-NIL, and NB. The data are the means ± s.d.s (25 plants × 3 repeats). The different letters indicate significant differences (P < 0.05, Tukey’s honestly significant difference (HSD) test). (**d**) Candidate region of the *RBG1* locus reported previously^29^ and graphical genotypes (left) and phenotypes (right) of plants containing recombinant DNA between marker loci RM24930 and RM24944. CEN indicates the centromere. (left) Four recombinants were selected from 3252 F_2_ plants derived from KO × SL535, and these recombinants have segments from chromosome 10 of NB. Black denotes regions homozygous for NB marker alleles; white denotes regions homozygous for KO marker alleles. (right) BSR severity of the recombinant lines KO and SL535. The data are the means ± s.d.s (15 plants × 3 repeats) (Student’s *t* test). (**e**) Physical map of the region around *RBG1* on chromosome 10 and sequence variations in KO, NB and Mut-W56* in the putative ORF detected in the candidate region at the *RBG1* locus (LOC_Os10g04000). *aa* amino acids, *ATG* initiation codon, *TAG and TGA* stop codons.

To clarify the effects of *RBG1res* on resistance to BSR, we developed a homozygous NIL for *RBG1res* in the KO genetic background (RBG1res-NIL) (Fig. 1a). RBG1res-NIL showed a higher level of resistance to BSR than did KO, although the level of resistance of RBG1res-NIL was not as high as that of NB (Fig. 1b,c). RBG1res-NIL showed consistent resistance against all five tested strains of *B. glumae,* indicating that *RBG1res* could confer broad-spectrum resistance to *B. glumae* (Supplementary Fig. S1).

To analyse the *RBG1* gene, we delimited a candidate region within a 3.7-kb segment between marker loci CAPS24 and CAPS32 via fine genetic mapping (Fig. 1d). One putative open reading frame (ORF), LOC_Os10g04000, is annotated in this region (Rice Annotation Project Database (RAP-DB) https://rapdb.dna.affrc.go.jp) (Fig. 1e). This putative gene, a viable candidate for *RBG1*, is predicted to be a mitogen-activated protein kinase kinase kinase (*MAPKKK*) gene. A comparison of the sequences of this ORF in NB and KO revealed three nucleotide differences, all of which were nonsynonymous substitutions in NB relative to KO: A343G, G390T, and T979G (Fig. 1e and Supplementary Fig. S2).

**Figure 2:**
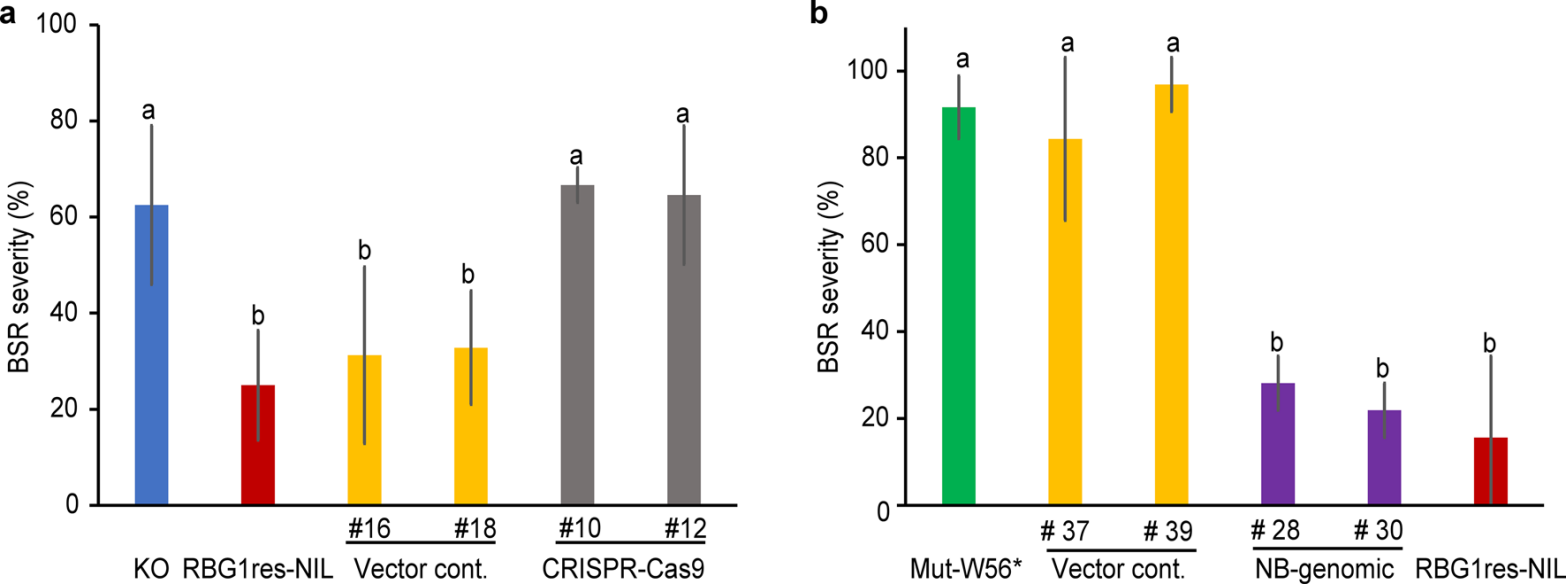
Assessment of BSR severity in transgenic lines. (**a**) Comparison of BSR severity in KO, RBG1res-NIL, and CRISPR/Cas9 knockout lines of RBG1res-NIL. Vector cont. refers to RBG1res-NIL transformed with an empty pZDgRNA binary vector. The data are the means ± s.d.s (8 plants × 4 repeats). The different letters indicate significant differences (P < 0.05, Tukey’s HSD test). (**b**) Comparison of BSR severity in *RBG1* transgenic plants. Mut-W56*, an *RBG1* knockout mutant, is the genetic background of the transgenic plants. NB-genomic (#28 and #30) and Vector cont. (#37 and # 39) are transgenic lines containing a single copy of the 5.5-kb *RBG1* genomic fragment from NB or the empty pPZP2H-lac binary vector, respectively. The data are the means ± s.d.s (8 plants × 4 repeats). The different letters indicate significant differences (P < 0.05, Tukey’s HSD test).

To determine whether *RBG1res* regulates resistance to BSR, knockout lines of *RBG1res* were created via the clustered regularly interspaced short palindromic repeat (CRISPR)/CRISPR-associated protein 9 (Cas9) system (Supplementary Fig. S2a). Because other QTLs for resistance to BSR in the NB genetic background may have masked the effect of *RBG1res*, we created CRISPR/Cas9 lines in RBG1res-NIL, which has a KO genetic background. The knockout lines created in the genetic background of RBG1res-NIL displayed more severe BSR symptoms than did RBG1res-NIL transformed with an empty vector (Fig. 2a). By using M_2_ lines of KO mutagenized with *N*-methyl-*N*-nitrosourea constructed according to the method described previously^31^, we identified a knockout mutant (Mut-W56*) in which a tryptophan residue at position 56 was changed, resulting in a stop codon that produces a truncated protein (5.5 kD) (Fig. 1e and Supplementary Fig. S2b). Transgenic Mut-W56* plants that carried a 5.5-kb genomic DNA fragment from NB (Fig. 1e), which contained the entire LOC_Os10g04000 ORF (NB genomic), showed decreased BSR severity compared to those plants transformed with an empty vector (Fig. 2b and Supplementary Fig. S3). Therefore, we concluded that the resistance to BSR of NB was provided by the NB allele at LOC_Os10g04000.

### *RBG1res* encodes a kinase that functions upstream of OsMKK3

**Figure 3:**
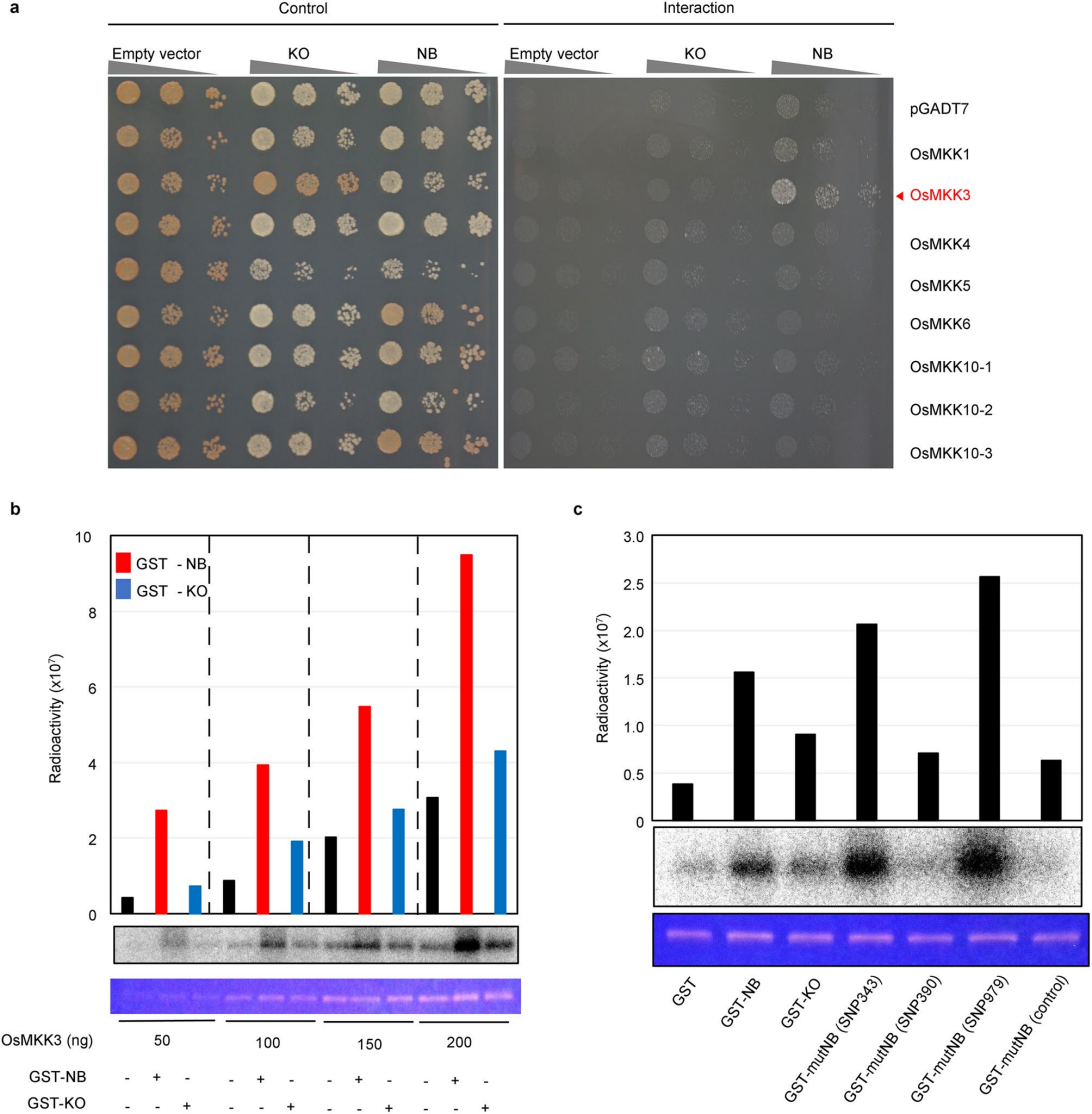
RBG1 is an upstream kinase of OsMKK3. (**a**) Y2H analysis of RBG1. The interactions of RBG1 and several rice OsMKK proteins were examined. RBG1 protein from KO or NB was used as bait. pGADT7 (empty vector) or vectors encoding OsMKK1, OsMKK3, OsMKK4, OsMKK5, OsMKK6, OsMKK10-1, OsMKK10-2, or OsMKK10-3 were used as prey. (**b**) Phosphorylation of OsMKK3 by RBG1 proteins by in vitro kinase assay. GST-NB refers to GST-fused NB RBG1; GST-KO refers to GST-fused KO RBG1. GST-RBG1 proteins (10 ng) were incubated with OsMKK3 as the substrate (50, 100, 150, or 200 ng) in the kinase reaction mixture, and aliquots of the samples were separated by SDS‒PAGE and subjected to autoradiography. The phosphorylation of OsMKK3 was observed in response to the addition of RBG1 proteins of both the NB and the KO types, and radioactivity indicative of phosphorylation by GST-NB (red bars) and GST-KO (blue bars) is shown in the top panel. Autophosphorylation of OsMKK3 is shown as a black bar. Autoradiography of OsMKK3 is shown in the middle panel. Oriole staining of OsMKK3 is shown in the bottom panel. (**c**) Phosphorylation of OsMKK3 by RBG1 proteins by in vitro kinase assay. GST refers to the GST control; GST-NB refers to GST-fused NB RBG1; GST-KO refers to GST-fused KO RBG1; GST-mutNB (SNP343) refers to GST-fused RBG1 that has the A115T amino acid substitution (KO type) introduced into the NB type; GST-mutNB (SNP390) refers to GST-fused RBG1 that has the N130K amino acid substitution (KO type) introduced into the NB type; GST-mutNB (SNP979) refers to GST-fused RBG1 that has the G327C amino acid substitution (KO type) introduced into the NB type; GST-mutNB (control) refers to GST-fused RBG1 that has the C524G amino acid substitution introduced into the NB type to eliminate kinase activity. GST-RBG1 proteins (10 ng) were incubated with OsMKK3 (200 ng) as the substrate. Radioactivity indicative of phosphorylation is shown in the top panel. Autoradiography of OsMKK3 is shown in the middle panel. Oriole staining of OsMKK3 is shown in the bottom panel.

*RBG1res* was predicted to be a *MAPKKK* gene, and RBG1 homologues were discovered in rice, Arabidopsis, grape, and sorghum by the use of the SALAD database (http://salad.dna.affrc.go.jp/salad/) (Supplementary Fig. S4). Phylogenetic analysis also showed that the sequences of several *MAPKKK* genes in rice were highly similar to that of *RBG1res*^32^ (Supplementary Fig. S5). To confirm the interactions between RBG1 and OsMKK proteins, we conducted yeast two-hybrid (Y2H) assays. We tested several OsMKKs and found that only OsMKK3 interacted with RBG1 from NB only (Fig. 3a).

To evaluate whether RBG1 is an upstream kinase of OsMKK3, we carried out in vitro kinase assays. Phosphorylation of OsMKK3 was observed by RBG1 from NB and KO; however, the phosphorylation of OsMKK3 was greater by RBG1 from NB than by RBG1 from KO (Fig. 3b and Supplementary Fig. S6).

To identify which of the sequence polymorphism(s) in *RBG1* (Fig. 1e) is responsible for the difference in resistance between KO and NB, we constructed three recombinant forms of RBG1: (i) GST-mutNB (SNP343), a GST-fused RBG1 protein that has the A115T amino acid substitution (KO type) introduced into the NB type; (ii) GST-mutNB (SNP390), a GST-fused RBG1 protein that has the N130K amino acid substitution (KO type) introduced into the NB type; and (iii) GST-mutNB (SNP979), a GST-fused RBG1 protein that has the G327C amino acid substitution (KO type) introduced into the NB type. Recombinant forms of RBG1 with either the SNP343 or SNP979 substitution showed slightly higher kinase activity than did the wild-type (WT) protein of NB (Fig. 3c and Supplementary Fig. S7). In contrast, the recombinant form of RBG1 with the SNP390 substitution showed much lower kinase activity than did the WT protein of NB. These results suggest that the G390T substitution, resulting in the substitution of lysine with asparagine in the NB-type RBG1 protein, is essential for kinase activity on OsMKK3.

### *RBG1res* confers resistance to *B. glumae* via negative regulation of ABA

**Figure 4:**
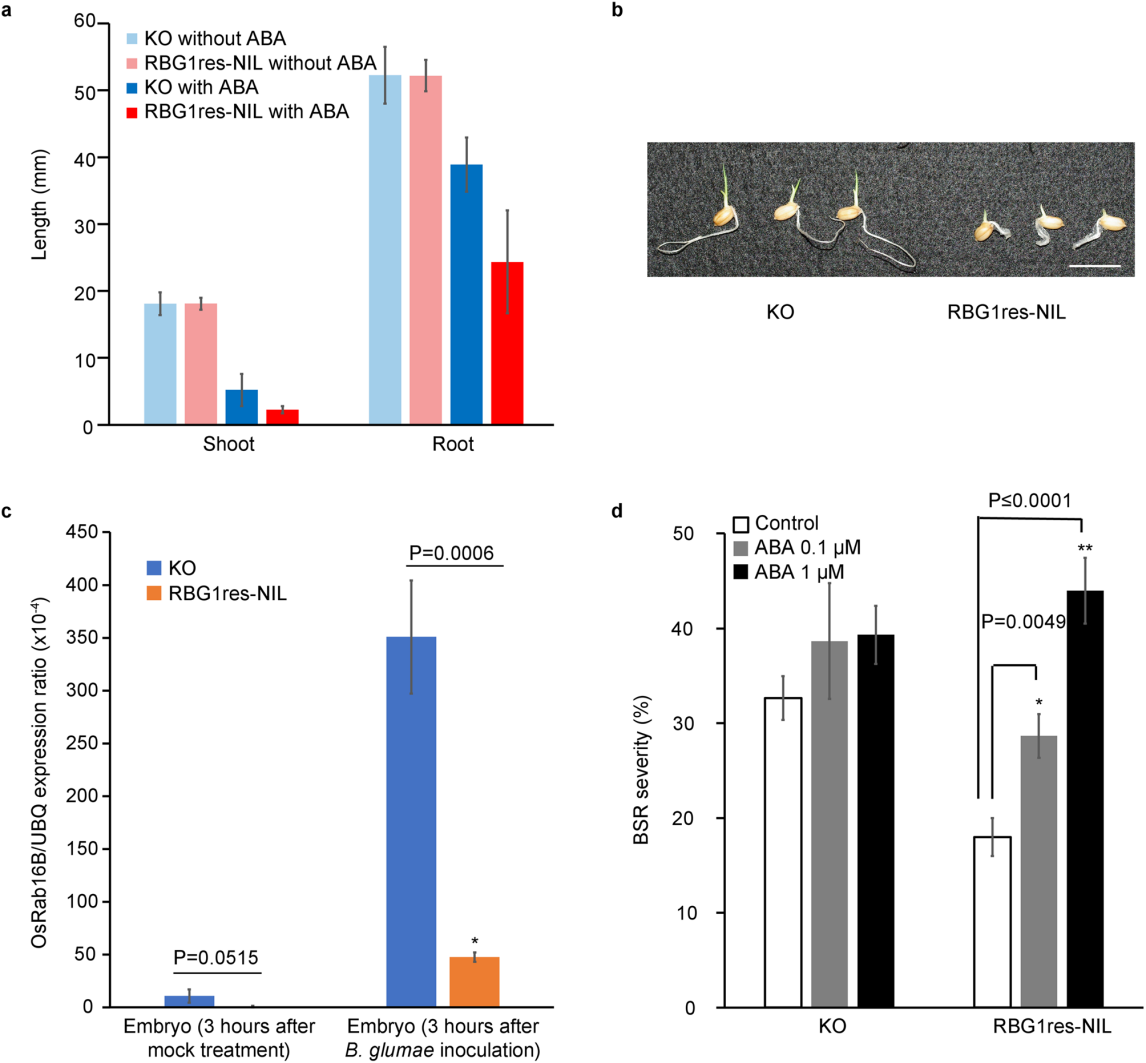
*RBG1res* confers resistance to *Burkholderia glumae* via negative regulation of ABA. (**a**) Comparison of the germination response between RBG1res-NIL and KO after treatment with a low concentration of ABA. The shoot and root lengths were measured at 3 days after planting seeds on media supplemented with ABA (1 μM). The data are the shown as the means ± s.d.s; n = 15 (Student’s *t* test). (**b**) Image of seeds at 3 days after treatment on media containing a low concentration of ABA (1 μM). The scale bar represents 1 cm. (**c**) Comparison of *OsRab16B* expression in embryos of KO and RBG1res-NIL after inoculation with *Burkholderia glumae* (*B. glumae*). *OsRab16B* is an ABA-inducible gene^34^, and the expression of *OsRab16B* was normalized to the expression of the ubiquitin gene. The data are the means ± s.d.s; n = 3. (Student’s *t* test). (**d**) BSR severity in KO and RBG1res-NIL after treatment with ABA. The seeds were first inoculated with *B. glumae*, after which ABA or water (control) was sprayed onto 4-day-old seedlings. BSR severity was measured at 3 days after ABA treatment. The data are the means ± s.d.s (25 plants × 3 repeats) (Student’s *t* test).

Because MKK3 in *Arabidopsis* was reported to be involved in ABA signalling^33^, we compared the responses to ABA between RBG1res-NIL and KO. Seeds were sown on media supplemented with low concentration of ABA, and growth was compared between the two lines. After 3 days of treatment, the shoots and roots of RBG1res-NIL were significantly shorter than those of KO (Fig. 4a,b). These results suggest that the response of germinating RBG1res-NIL seedlings to exogenous ABA is different from that of germinating KO seedlings. We then monitored ABA responses in KO and RBG1res-NIL by the use of *OsRab16B*, a well-characterized ABA-inducible gene^34^. We performed qRT‒PCR to investigate whether the expression of *OsRab16B* was induced after inoculation with *B. glumae* and found that the degree of induction of *OsRab16B* in the embryos of KO at 3 h after inoculation was much higher than that of RBG1res-NIL (Fig. 4c). Thus, we considered the possibility that the resistance observed in RBG1res-NIL is caused by negative regulation of the ABA response after inoculation. To test this possibility, we measured the BSR severity in KO and RBG1res-NIL after treatment with ABA. By spraying ABA on the inoculated seedlings, we found that the BSR severity increased in RBG1res-NIL but not in KO (Fig. 4d). These results indicated that *RBG1res* conferred resistance to *B. glumae* via negative regulation of ABA. Previous investigations have shown that MKK3 also functions in some signalling pathways related to the response to abiotic stresses^35,36^. Therefore, we evaluated the responses of RBG1res-NIL and KO to abiotic stress and found that *RBG1res* does not affect tolerance to low or high temperature (Supplementary Fig. S8a,b), salt (Supplementary Fig. S8c), or drought (Supplementary Fig. S8d,e).

### Natural variation in *RBG1* and the evolution of the NB allele

**Figure 5:**
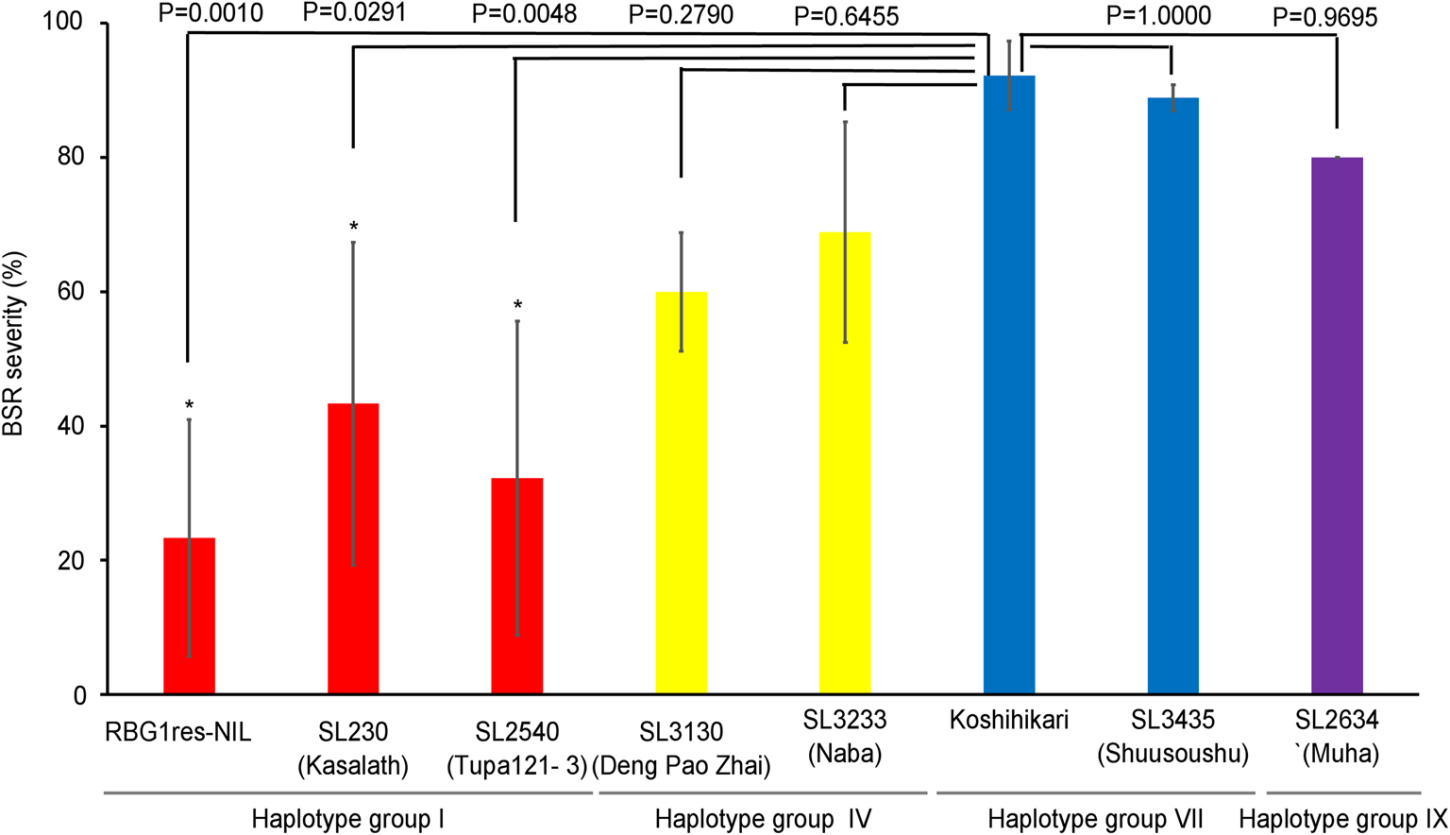
Comparison of BSR severity in RBG1res-NIL, KO, and representative CSSLs. SL230 (Kasalath) is a CSSL in which part of the short arm of chromosome 10 of KO is substituted with the corresponding segment of Kasalath. The Kasalath allele at *RBG1* belongs to haplotype group I (Supplementary Table S1), as do the *RBG1* alleles in RBG1res-NIL and SL2540 (Tupa 121–3). The *RBG1* alleles in SL3130 (Deng Pao Zhai) and SL3233 (Naba) belong to haplotype group IV, those in KO and SL3435 (Shuusoushu) belong to group VII, and those in SL2634 (Muha) belong to group IX. The data are the means ± s. d; n = 3 blocks (15 plants per block). Each line of haplotype group I differed from the susceptible control (KO) significantly in terms of BSR severity at the 5% level according to Dunnett’s test.

Sequence investigation of the *RBG1* transcribed regions of 70 rice cultivars revealed 11 haplotypes (Supplementary Table S1). The Type I haplotype group, which included NB, consisted of several cultivars originating from India and its neighbouring countries. Combining the resistance information in our previous study of these cultivars^28^ with the sequence information obtained here, we found no correlation between haplotype pattern and BSR resistance, suggesting that other QTLs for resistance to BSR in these genetic backgrounds may have masked the effect of *RBG1*. By using CSSLs (KO background) that we previously developed from diverse rice accessions with widespread geographic distribution^37^, we were able to reveal the effects of allelic variations of *RBG1* in a uniform genetic background, which eliminated the effects of QTLs other than *RBG1*. The CSSLs with the Type I haplotype had significantly less severe BSR than did KO, whereas the BSR resistance of those with other haplotypes did not differ significantly from that of KO (Fig. 5). Because the G390T substitution in *RBG1* was found to be essential for resistance to BSR (Fig. 3c), we compared single-nucleotide polymorphisms (SNPs) at *RBG1* position 390 among 425 accessions of *Oryza rufipogon*, a recent ancestor of *O. sativa*, within the OryzaGenome database (http://viewer.shigen.info/oryzagenome/mapview/Top.do), and 25 accessions had the same SNP as NB (Supplementary Table S2). These results suggest that the G390T substitution in *RBG1* first occurred in *O. rufipogon* and has been selected as an advantageous variant due to its resistant phenotype against *B. glumae* and that this SNP now is present in several cultivars, including NB, through evolution and selection.

### Expression analysis of *RBG1 *variants and effect of *RBG1res* on resistance to other pathogens

*RBG1* was expressed at the highest levels in the embryos, at low levels in the young leaves, and at barely detectable levels in the shoots, roots, leaf blades, and spikelets of KO and RBG1res-NIL (Supplementary Fig. S9a). The expression patterns in KO and RBG1res-NIL were very similar.

*MKK3* was reported to regulate preharvest sprouting in barley and wheat^38,39^. To investigate whether *RBG1* is also involved in preharvest sprouting, we analysed its expression in seeds after flowering. *RBG1* mRNA was detected in the embryo and endosperm 42 days after flowering (DAF), whereas it was hardly detected in either tissue at 7, 14, or 28 DAF from KO and RBG1res-NIL (Supplementary Fig. S9b). The expression patterns in KO and RBG1res-NIL were similar, as were the levels of seed dormancy in a germination test (Supplementary Fig. S9c). The germination rates of seeds sampled at various time points after heading were nearly the same in KO and RBG1res-NIL (Supplementary Fig. S9d). These findings suggested that *RBG1* was induced specifically in mature seeds from KO and RBG1res-NIL and that the differences between the KO and NB alleles of *RBG1* did not influence seed dormancy or preharvest sprouting. We carried out qRT‒PCR to monitor responses to *B. glumae* in KO and RBG1res-NIL and found that *RBG1* expression in both lines was almost completely abolished after inoculation with either *B. glumae* inoculant or water (Supplementary Fig. S9e). Moreover, no differences in the expression patterns of pathogenesis-related (PR) genes (*PR2* and *WRKY45*) between KO and RBG1res-NIL after inoculation with *B. glumae* were observed (Supplementary Fig. S10).

**Figure 6:**
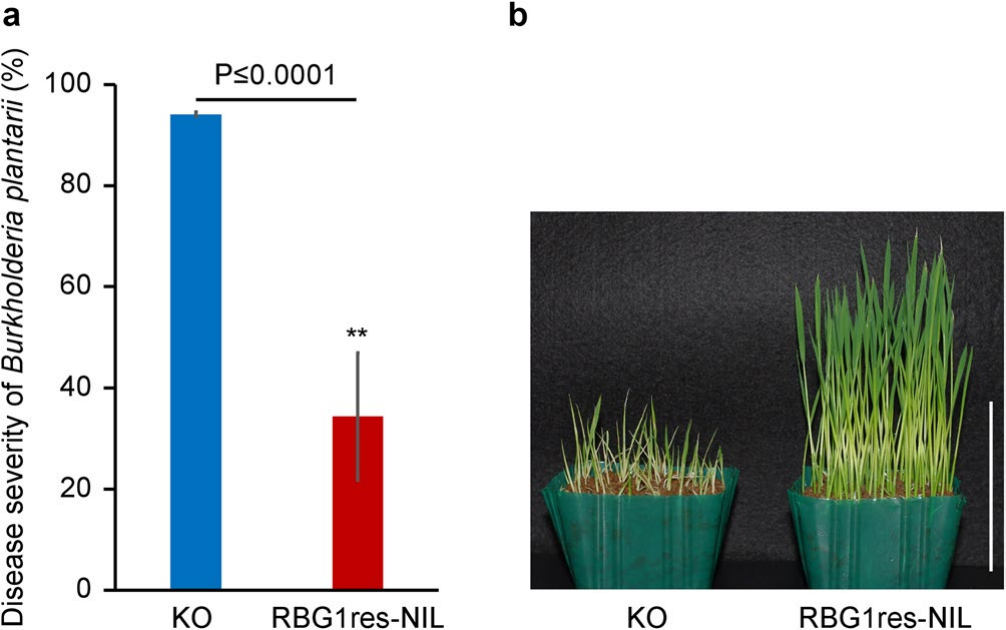
Resistance to bacterial seedling blight caused by *Burkholderia plantarii* in KO and RBG1res-NIL. (**a**) Disease severity of *Burkholderia plantarii* (*B. plantarii*). The data are the means ± s.d.s; n = 4 blocks (100 plants per block). (Student’s *t* test). (**b**) Phenotypes of KO and RBG1res-NIL 7 days after sowing inoculated seeds. The scale bar represents 6 cm.

Because *RBG1* is specifically induced in mature seeds from KO and RBG1res-NIL, we speculated that RBG1res-NIL at the germination stage might also be resistant to pathogens other than *B. glumae*. We compared the resistance to bacterial seedling blight caused by *B. plantarii* between RBG1res-NIL and KO and found that RBG1res-NIL showed less severe *B. plantarii* symptoms at the time of germination (Fig. 6). We concluded that *RBG1res* may confer broad resistance to germinating seeds, although further analysis is necessary to elucidate the underlying mechanism of resistance provided by *RBG1res* against *B. plantarii*.

Since *RBG1* was found to be exclusively induced in mature seeds, we evaluated the disease severity of mature plants in the field and found that the disease severity in the panicles caused by *B. glumae* was not significantly different between KO and RBG1res-NIL (Supplementary Fig. S11a,b). Moreover, RBG1res-NIL was not found to be more resistant than KO was against other pathogens, such as *Pyricularia oryzae* (formerly named *Magnaporthe grisea*), the causal agent of leaf blast of rice, or *Xanthomonas oryzae* pv. *oryzae* (*Xoo*), the causal agent of bacterial blight of rice (Supplementary Fig. S11c,d).

## Discussion

Here, we revealed that *RBG1res* encodes a *MAPKKK* involved in resistance to BSR. In a previous report, 75 *MAPKKKs* were identified via computational analysis of sequenced rice genomes and could be classified into three subgroups: Rafs, ZIKs, and MEKKs^40^. In that report, *RBG1* was registered as *MAPKKK67* and classified into the MEKK subfamily. Many previous studies support the idea that MEKK-like members function as MAPKKKs upstream of MAPKKs in plant MAPK cascades^41^. Our results showed that RBG1 phosphorylates OsMKK3, regulating its activity. Plant MAPKKs can be classified into four different groups (A–D), and Group B MAPKKs include *Arabidopsis* MKK3^41^. As determined by a comparison of the phenotypes of RBG1res-NIL and KO, *RBG1res* did not affect tolerance to low or high temperature, salt, or drought. Therefore, it remains unknown how OsMKK3 phosphorylated by RBG1 functions in the stress response-related signalling pathway*.* It is first necessary to reveal the steps downstream of the RBG1-OsMKK3 cascade.

Our results show that the three SNPs (A343G, G390T, and T979G) between KO and NB cause nonsynonymous substitutions and that the G390T substitution within RBG1 in NB is essential for its increased kinase activity on substrates, including OsMKK3. According to the alignment of kinase domains of MAPKKK-related proteins from *Nicotiana tabacum*, *Arabidopsis thaliana*, and *Oryza sativa*^32^, all 11 kinase subdomains were found to be common. Among those subdomains, the catalytic loop HRDIKXX in subdomain VI is essential for substrate binding and catalysis and is conserved across all MAPKKKs^42^. The position corresponding to G390T is just before the catalytic loop. Therefore, this substitution in NB (lysine to asparagine) is likely to induce strong kinase activity and confers resistance to BSR. We compared SNPs at position 390 in *RBG1* among the 425 accessions of *O. rufipogon*, and the SNP in only 25 accessions was the same as that in NB. Sequence investigation of the *RBG1* transcribed regions of rice cultivars revealed 11 haplotypes, and the CSSLs whose type of *RBG1* was the same as that of NB (Type I haplotype) displayed significantly less severe BSR than did those with other haplotypes. These results suggest that the *RBG1res* haplotype originated in *O. rufipogon* and that several cultivars, including NB, were selected for their resistance to *B. glumae*.

Two types of defence mechanisms in plants are well known: pathogen-associated molecular pattern (PAMP)-triggered immunity (PTI) and effector-triggered immunity (ETI)^43^. MAPK cascades are the general components involving plant defence signalling, mainly in the PTI signalling pathway^41,43^. *WRKY45* regulates disease resistance mediated by the salicylic acid (SA) signalling pathway in rice and is reportedly regulated by the OsMKK10-2–OsMPK6 cascade^44^. In the present study, no difference in the expression pattern of *WRKY45* was observed between RBG1res-NIL and KO after inoculation with *B. glumae*. Taken together with the result that the RBG1 protein functions as an upstream kinase of only OsMKK3 and not OsMKK10-2, these findings suggest that the signalling pathway of *RBG1* is different from the SA signalling pathway related to *WRKY45*. PR genes are known to be activated mainly in ETI^43^, and no difference in the expression pattern of *PR2* was observed between RBG1res-NIL and KO after inoculation with *B. glumae*. Since no differences in expression of *WRKY45* and *PR2* between susceptible and resistant lines related to *RBG1* was observed, it is expected that other resistance mechanisms associated with ETI and PTI could be involved in *RBG1res*. In contrast to *RBG1res* being the first QTL identified for resistance to *B. glumae*, overexpression of a receptor-like cytoplasmic kinase, which was named *BSR1,* was found to induce resistance to *B. glumae*^45,46^. *BSR1* was reported to confer broad-spectrum resistance to *Xoo, P. oryzae,* and *Cochliobolus miyabeanus*, in addition to *B. glumae*^45^. In contrast to lines containing *BSR1*, RBG1res-NIL was discovered to be resistant to *B. glumae* and *B. plantarii* but not to *Xoo* or *P. oryzae*. Further investigations are needed to clarify whether the differences between *BSR1* and *RBG1* result from differences in expression patterns or in protein function. As mentioned above, *B. glumae* causes both BSR and BGR in rice. Because *RBG1res* is induced specifically in mature seeds (42 DAF), *RBG1res* may not confer resistance to BGR.

Although *MKK3* was reported to regulate preharvest sprouting in barley and wheat^38,39^, the germination rates of seeds sampled at various time points after heading were similar between RBG1res-NIL and KO. These differences between *RBG1* in rice and *MKK3* in barley and wheat may be the result of the unique expression pattern of *RBG1*. *TaMKK3-A*, a causal gene for seed dormancy in wheat, is expressed at relatively constant levels in all organs examined^39^. Likely because *RBG1res* was shown to be expressed specifically in mature seeds, *RBG1res* did not influence preharvest sprouting. Compared with barley and wheat, rice is commonly cultivated in the summer season in warm areas, and pathogen attacks are severe at the germination stage. Thus, it is possible that resistance during germination is particularly important in rice and that *RBG1res*, which is expressed specifically in mature seeds, was selected during the process of evolution.

The response to ABA differed between RBG1res-NIL and KO. RBG1res-NIL appeared to be more sensitive to exogenous ABA than did KO during germination. These results suggested that *RBG1res* affects ABA signalling. Interestingly, transgenic rice expressing *Lr34res* also exhibited a hypersensitive response to exogenous ABA during germination^47^. *Lr34res* is a well known resistance gene against multiple fungal pathogens in wheat. Because *Lr34res* was discovered to function as an ABA transporter and because ABA accumulation was observed in *Lr34res*-expressing transgenic rice lines^47^, we supposed that there may be a similar mechanism between *RBG1res* and *Lr34res.* It is necessary to clarify whether ABA accumulation occurs in RBG1res-NIL. Moreover, *WRKY45* was not differentially expressed according to the RNA sequencing data of *Lr34res*-expressing transgenic rice lines^47^. Since no differential expression of *WRKY45* between susceptible and resistant lines related to *RBG1* was observed, we hypothesized that *RBG1res* and *Lr34res* trigger similar resistance mechanisms initiated by ABA signalling. As determined by a comparison of the phenotypes of RBG1res-NIL and KO, *RBG1res* did not affect ABA-mediated physiological changes such as those in response to drought stress. Likely because e*RBG1res* was shown to be expressed specifically in mature seeds, we supposed that *RBG1res* did not influence the response to drought stress after the germination stage. Because *OsRab16B* was reported to be ABA inducible^34^, we investigated whether the expression of *OsRab16B* was induced after inoculation with *B. glumae*. The degree of induction of *OsRab16B* in the embryos at 3 h after inoculation was much higher in KO than in RBG1res-NIL. Therefore, the resistant phenotype of RBG1res-NIL may be caused by suppression of ABA after inoculation. To test the possibility that suppression of ABA leads to resistance, we measured BSR severity in KO and RBG1res-NIL after treatment with ABA. When ABA was sprayed onto inoculated seedlings, the BSR severity increased in RBG1res-NIL but not in KO. These results indicate that *RBG1res* confers resistance to *B. glumae* via negative regulation of ABA. The role of ABA in disease resistance depends on the type of pathogen and the timing of the defence response^48^. According to Sigh et al.^43^, ABA, which is often related to abiotic stress tolerance, is a negative regulator of the biotic stress response. Exogenous applications of ABA suppress resistance, and ABA biosynthesis inhibitors reduce susceptibility against rice blast^48–50^. In contrast, it has been shown that *P. oryzae* produces and secretes ABA during the infection process to induce susceptibility^51^. Therefore, it is unknown whether *RBG1res* confers resistance via negative regulation of ABA biosynthesis or by its effects on the ABA secreted from bacterial pathogens. Further analysis is needed to clarify how *RBG1res* regulate ABA. In conclusion, we emphasize that *RBG1res* is the first identified and unique QTL for resistance to *Burkholderia* strains, encodes a *MAPKKK* gene and confers resistance by negative regulation of ABA.

## Methods

### Plant materials

NB is a traditional lowland indica cultivar that originated in India and is resistant to BSR caused by *Burkholderia glumae.* KO is a modern lowland rice cultivar released in Japan and is susceptible to BSR^27^. To analyse *RBG1res*, by crossing SL535 with KO and using marker-assisted selection to remove nontarget DNA regions, we successfully developed a NIL homozygous for *RBG1res*. The resulting RBG1res-NIL contains approximately 380 kb from NB on the short arm of chromosome 10 (between simple sequence repeat (SSR) markers RM474 and RM7361-1; Supplementary Table S3). By screening 3072 M_2_ lines of KO mutagenized with *N*-methyl-*N*-nitrosourea according to a method described previously^31^, we identified a null mutant (Mut-W56*) whose sequence encoding tryptophan at position 56 was changed such that a stop codon was introduced that produces a truncated protein (5.5 kD). Genomic DNA of the M_2_ plants was screened with the NB51 primer set listed in Supplementary Table S3 by the targeting induced local lesions in genomes (TILLING) method as described earlier^52^. All of the experimental research and field studies on plants (either cultivated or wild), including transgenic plant materials, complied with relevant institutional, national, and international guidelines and legislation.

### Assessment of resistance to *B. glumae and B. plantarii*

The bacterial strain used in this study, except those referenced in Fig. 6, Supplementary Figs. S1 and S11, was *B. glumae* MAFF301682 (MAFF designates strains from the culture collection of the National Agriculture and Food Research Organization (NARO) Genebank, formerly the culture collection of the Ministry of Agriculture, Forestry and Fisheries, Japan). Bacterial inocula were incubated on Luria–Bertani (LB) media with 2% agar at 28 °C for 4 days and then suspended in sterilized, deionized water at a concentration of 10^8^ CFU/ml. The rice seeds were sterilized by soaking in a chlorine bleach solution (available chlorine 2.5%) for 30 min, rinsed carefully with sterilized water, and then soaked in sterilized water for 3 days in a plant growth chamber at 28 °C. The sterilized seeds were subsequently placed in a freshly prepared bacterial suspension and held under vacuum (0.2 MPa) for 3 min. The inoculated seeds were dried for 2 h, sown in sterilized soil (Bonsol No. 2, Sumitomo Kagaku Kougyo, Osaka, Japan) and incubated in a growth chamber at 28 °C with 80% humidity under a 14-h photoperiod. The disease symptoms were measured 8 days after sowing on a scale of 1–3, where 1 = no symptoms, 2 = sheaths with reddish-brown lesions (mild infection), and 3 = necrotic seedlings or seeds that did not germinate (severe infection). The BSR severity was calculated from these scores as follows:

$$ {\text{BSR}}\,{\text{severity }}\left( \% \right) \, = \, \left( {N_{{3}} {-}N_{{1}} {-}N_{{2}} /{2}} \right) \times {1}00/N_{{3}} , $$

where *N*_1_ = number of seedlings with a score of 1, *N*_2_ = number of seedlings with a score of 2, and *N*_3_ = number of seeds per replication. There were three or four replications per inoculation. As a control, we germinated uninoculated seeds and confirmed that the average germination rate was > 90%. The bacterial strain for evaluation of resistance to bacterial seedling blight was *B. plantarii* MAFF301723. The method for inoculating seeds was the same as that used for *B. glumae*. Five inoculated seeds and 95 uninoculated seeds were sown in sterilized soil in the same cell tray, and the disease severity of *B. plantarii* (Fig. 6) was calculated as described above. The bacterial strain shown in Supplementary Fig. S11 was *B. glumae* MAFF302744, and the panicle disease severity assay was conducted according to a previously described metohd^53^.

### Assessment of resistance to *P. oryzae *and *Xoo*

Resistance to *P. oryzae* (formerly named *M. grisea*) was analysed in an experimental field at the Institute of Crop Science (NICS). The field had high levels of *P. oryzae* infection, and the major race was 037.3. The lesion area (percentage of total leaf area) of 60-day-old plants was measured according to the previously described methods^54,55^. For the assessment of resistance to bacterial blight caused by *X. oryzae* pv. *oryzae*, flag leaves of 14-week-old plants in an experimental paddy field were inoculated according to a previously described clipping method^56^. A bacterial suspension (OD_660_ = 0.1; 2 × 10^9^ CFU/ml) was prepared, and lesion lengths were scored 20 days after inoculation with virulent *Xoo* race 2 strains (T7147, MAFF311019).

### High-resolution mapping of *RBG1res*

We mapped *RBG1* (formerly named *qRBS1)* between SSR markers RM24930 and RM24944 (Table S3) in a previous study^29^. For the high-resolution mapping of *RBG1res*, we used 3252 F_2_ plants generated from a cross between SL535 and KO and selected 37 plants in which recombination had occurred within the region containing *RBG1res*. These plants were self-pollinated, and the progeny were analysed for positional cloning of *RBG1res*.

### Vector construction and plant transformation

For the complementation test, a 5.5-kb genomic fragment of NB was amplified by PCR with the “RBG1-genomic” primer pair listed in Supplementary Table S3 and then cloned and inserted into a pPZP2H-lac binary vector. The sequence of the clone was confirmed. For genome editing, the CRISPR/Cas9 cleavage site of *RBG1res* was prepared using CRISPR-P 2.0 (https://cbi.hzau.edu.cn/CRISPR2/), and the vectors were constructed according to a previously published method^57^. We cloned the guide RNA (gRNA) expression cassettes and inserted them into a pZDgRNA binary vector by cleavage with AscI and PacI. The primers used for this experiment are shown in Supplementary Table S3.

The resulting constructs were introduced into *Agrobacterium tumefaciens* strain EHA101 by electroporation. *Agrobacterium*-mediated rice transformation was then performed as described previously^58,59^. A single copy was selected using the hygromycin phosphotransferase gene by segregation among the progeny. Control plants were generated by introducing an empty vector.

### RNA extraction and analysis of expression by qRT‒PCR

Total RNA was extracted from various tissues (embryo, endosperm, shoot, root, young leaf, leaf blade, spikelet and ovary tissues) of KO, RBG1res-NIL and inoculated plants using an RNeasy Plant Mini Kit (Qiagen) and RNA suisui (Rizo, Inc., Tsukuba, Japan). RNA suisui was used for isolation of RNA from embryos and endosperm, whereas the RNeasy Plant Mini Kit was used for all other tissues. First-strand cDNA was synthesized using SuperScript II Reverse Transcriptase (Invitrogen). Quantitative RT‒PCR using TaqMan probes was performed with specific primers and probes (Supplementary Table S2). The PCR conditions were 10 min at 95 °C followed by 50 cycles of 15 s at 95 °C followed by 1 min at 60 °C. Expression of the target genes was normalized to the expression of the ubiquitin gene. All the assays were performed at least three times. To compare the expression of *RBG1*, *WRKY45*, and *PR2* between KO and RBG1res-NIL after inoculation with *B. glumae*, both seed types were soaked in a bacterial inoculum suspension whose concentration was adjusted to 10^8^ CFU/ml with sterilized water and incubated for 2 days at 28 °C.

### Sequence alignment and phylogenetic tree construction

The genomic sequences corresponding to the transcribed regions of *RBG1* were amplified by PCR with three primer sets (“Sequence analysis”; Supplementary Table S2). The PCR products were sequenced by using a BigDye Terminator v.3.1 Cycle Sequencing Kit (Life Technologies). BLAST searches were conducted using the amino acid sequences of the kinase domain of RBG1 as queries in the SALAD database (https://salad.dna.affrc.go.jp/salad/). Using GENETYX v.12 (GENETYX Corp. Shibuya, Japan), we constructed a phylogenetic tree by the neighbour-joining method (1000 bootstrap replicates).

### Germination test

Panicles were sampled at 4, 6, 8, and 10 weeks after heading, and 50 seeds from each panicle were used for germination tests. The seeds were placed on filter paper in a petri dish, 20 ml of sterilized water was added, and the dishes were incubated in the dark at 30 °C for 7 days.

### Y2H assay

A bait construct was made by inserting a DNA fragment containing *RBG1*, which was amplified via PCR by using specific primers (Supplementary Table S2), into a pGBKT7 vector (Clontech) with EcoRI and PstI sites. Prey constructs were made by inserting DNA fragments encoding OsMKK1, OsMKK3, OsMKK4, OsMKK5, OsMKK6, OsMKK10-1, OsMKK10-2, and OsMKK10-3 (their sequence data were obtained from the Rice Annotation Project Database (RAP-DB)) into a pGADT7 vector by an in-fusion kit (Takara Bio). Y2H assays were conducted according to the manufacturer’s protocol (Clontech). The cells were assigned to SD minimal media (Clontech) lacking leucine and tryptophan and were subsequently streaked onto media lacking leucine, tryptophan, histidine, and adenine and supplemented with 5 mM 3-amino-1,2,4-triazole (3-AT). The plates were subsequently incubated at 30 °C for 3 days.

### In vitro kinase assay

The kinase reaction mixture consisted of buffer (50 mM Tris–HCl (pH 7.5), 2 mM EGTA, 20 mM MgCl_2_, 2 mM MnCl_2_, 1 mM dithiothreitol (DTT), 100 mM cold ATP, and 0.074 MBq (^32^P) ATP (3000 Ci/mmol)). The reaction temperature and time were 30 °C and 30 min, respectively. Glutathione S-transferase (GST) fusion RBG1 proteins and OsMKK3 were synthesized by CellFree Sciences, Co., Ltd (Yokohama, Japan). A GST-fused RBG1 protein (enzyme) and OsMKK3 (substrate) were added to the reaction mixture, and the samples were dissociated by SDS‒PAGE and subjected to autoradiography. The radioactivity was measured using a Typhoon FLA 9500 scanner. The volume of radioactivity, which was analysed by ImageQuant TL software (GE Healthcare Life Sciences), is the relative value by multiplication of signal intensity and pixel number.

### Statistical methods

The statistical analysis was conducted with JMP v.14.0.0. software (SAS Institute).

## Supplementary Information

Supplementary Tables.

Supplementary Figures.

## Data Availability

The datasets generated in this study will be available in the DNA Data Bank of Japan (DDBJ) repository (https://www.ddbj.nig.ac.jp). The DDBJ accession numbers for *RBG1* for NB and KO will be LC730906 and LC730907, respectively. According to the rules of DDBJ, the accession numbers are undisclosed until the acceptance of this report about *RBG1* in order not to leak the data to competitors. As a tentative measure, we added the sequence data to the Supplementary Information File.
