# Supplementary material for: A MAPKKK gene from rice, RBG1res, confers resistance to Burkholderia glumae through negative regulation of ABA
Source: Sci Rep. 2023 Mar 9;13:3947. doi: 10.1038/s41598-023-30471-9 (PMC9998638; doi:10.1038/s41598-023-30471-9)

**Supplementary Figure S1. BSR severity of ‘Koshihikari’ (KO) and RBG1res-NIL infected with five strains of *Burkholderia glumae*.** Five inoculated seeds and 95 uninoculated seeds were sown in the same cell tray in sterilized soil, and disease severity was measured at 7 days after sowing. Data are means ± s. d; n=4 blocks (100 plants per block). (Student’s *t*-test).


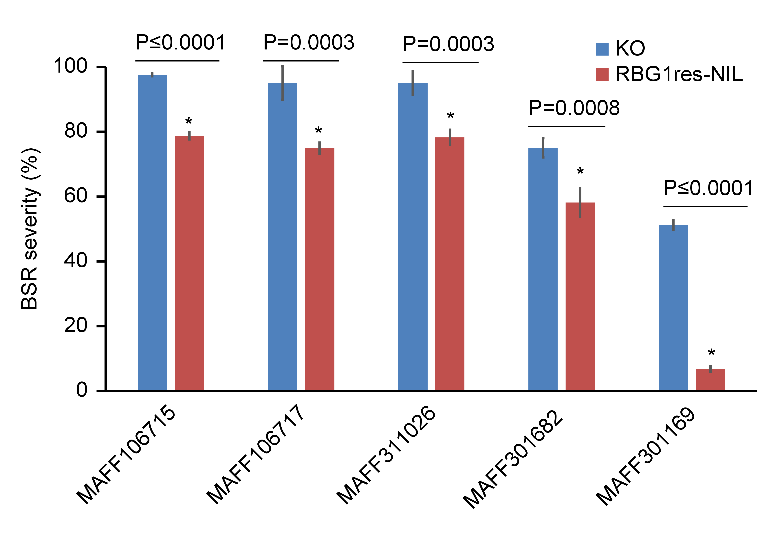


**Supplementary Figure S2. Amino acid sequences of *RBG1* in NB(a) and KO(b).**

The three blue squares in each panel indicate single-nucleotide polymorphisms (SNPs) between NB and KO. Purple circle indicates the single nucleotide deletion in the CRISPR-Cas9 lines. Red circle indicates the nucleotide, which changes to A in Mut-W56* resulting in a stop codon.


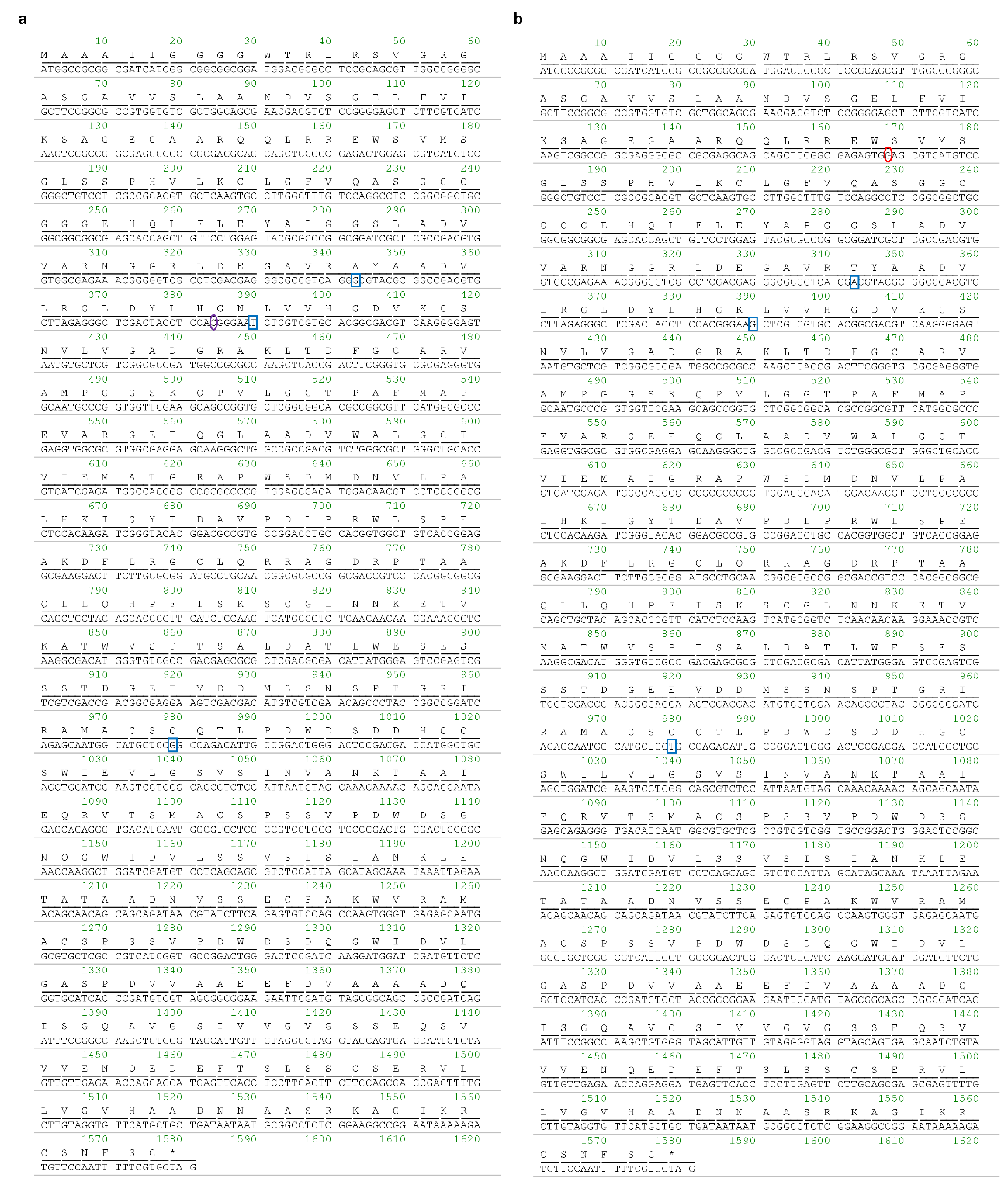


**Supplementary Figure S3.** **Differences in resistance to BSR caused by *B. glumae* between RBG1res-NIL, Mut-W56* and transgenic plants of *RBG1*.** The inoculated seedlings are representative phenotypes of Figure 2b. Mut-W56*, a *RBG1* knockout mutant, is the genetic background of the transgenic plants. NB-genomic (#30) and Vector cont. (#39) are transgenic lines containing a single copy of the 5.5-kb *RBG1* genomic fragment from NB or the empty pZDgRNA binary vector, respectively. Almost all of the seedlings of Mut-W56* and Vector cont. have sheaths with reddish-brown lesions. Photo shows seedlings 7 days after sowing inoculated seeds with bacterial suspension at a concentration of 10^8^ CFUs per ml. Scale bar represents 5 cm.


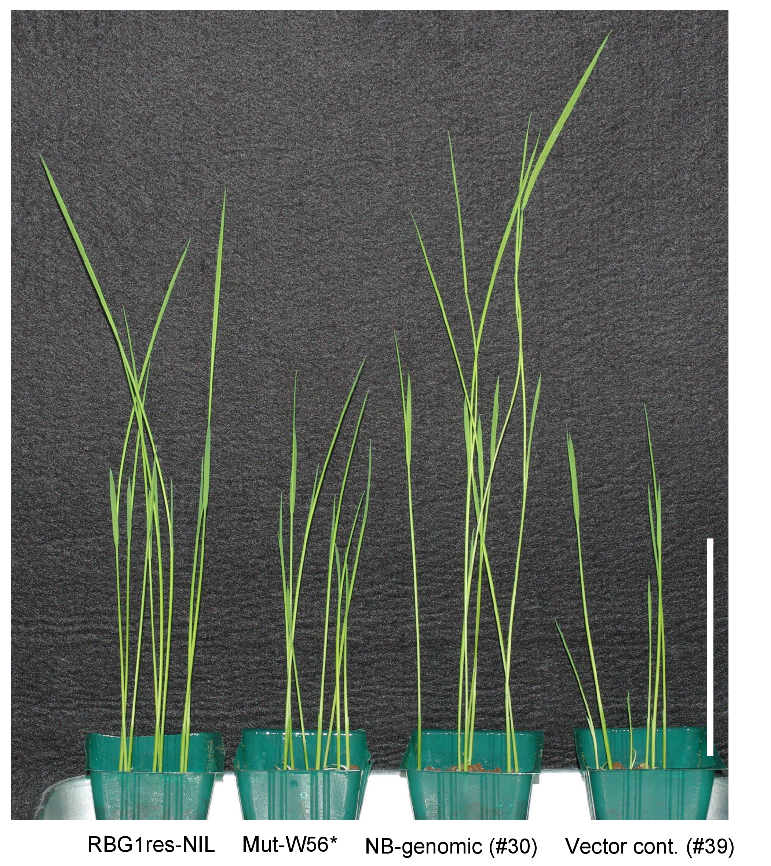


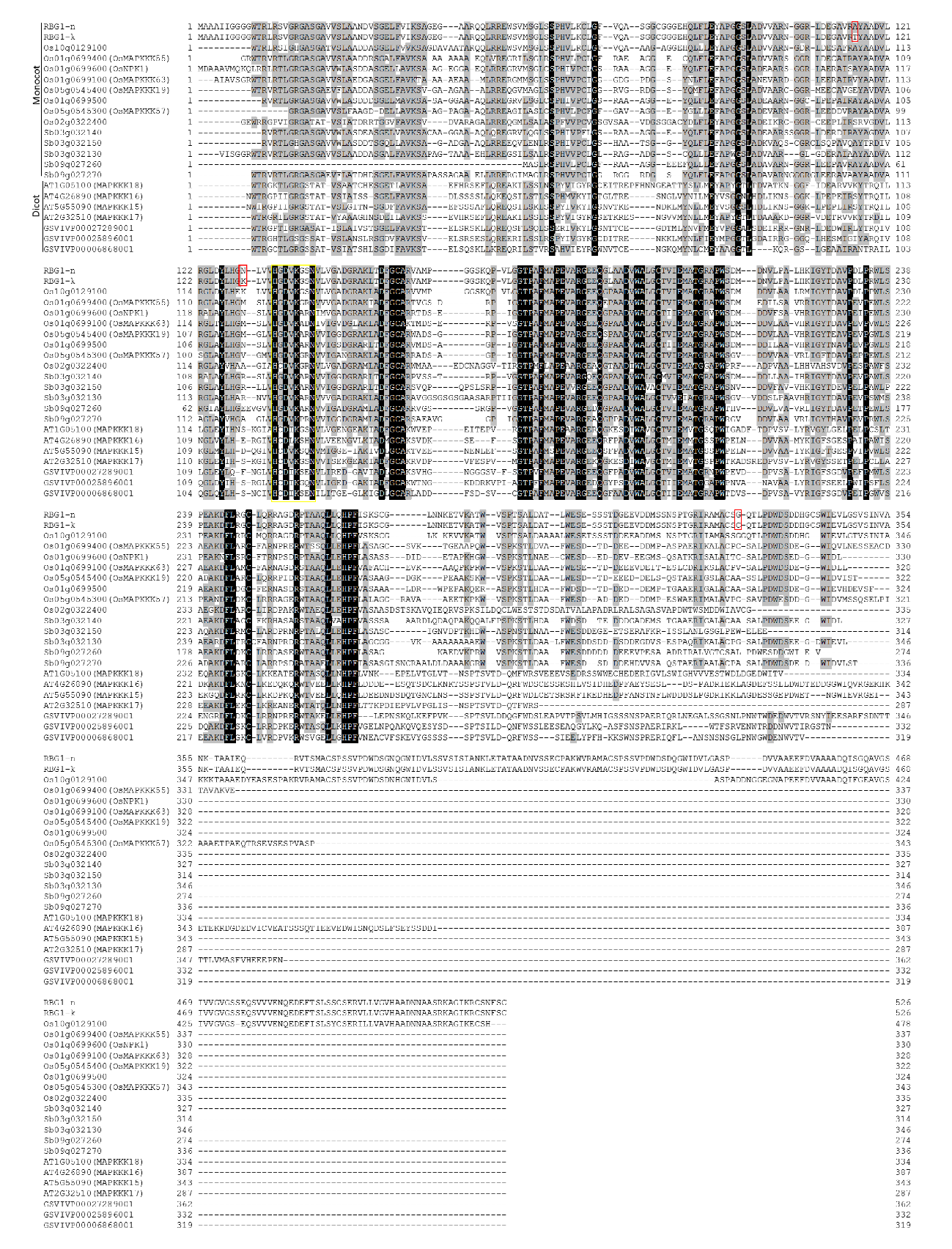
**Supplementary Figure S4. Comparison of deduced amino acid sequences between RBG1 and its homologs.** RBG1-n, RBG1 of ‘Nona Bokra’. RBG1-k, RBG1 of ‘Koshihikari’. Other sequences are from *Oryza sativa* (Os), *Sorghum bicolor* (Sb), *Arabidopsis thaliana* (AT), and *Vitis vinifera* (GSVIVP). *Shaded boxes* indicate residues that are identical in a majority of sequences. *Black boxes* indicate residues that are identical in all sequences. The three SNPs between ‘Nona Bokra’ and ‘Koshihikari’ are highlighted in *red boxes*. The conserved subdomain VI in MAPKKKs is shown in a *yellow box*.

**Supplementary Figure S5. Phylogeny of rice and *Arabidopsis* genes encoding a conserved kinase domain composed of 258 amino acids.** *RBG1* homologs were searched by BLAST using the SALAD database (http://salad.dna.affrc.go.jp/salad/). Sequences were aligned by GENETYX ver.12 and a phylogenetic tree was constructed using the neighbor-joining method. The number at each node represents the bootstrap value out of a total of 1000 bootstraps. Seventy-five MAPKKKs were identified by computational analysis of a sequenced rice genome of ‘Nipponbare’ ^33^, and *RBG1* was numbered as *MAPKKK67* in that analysis.


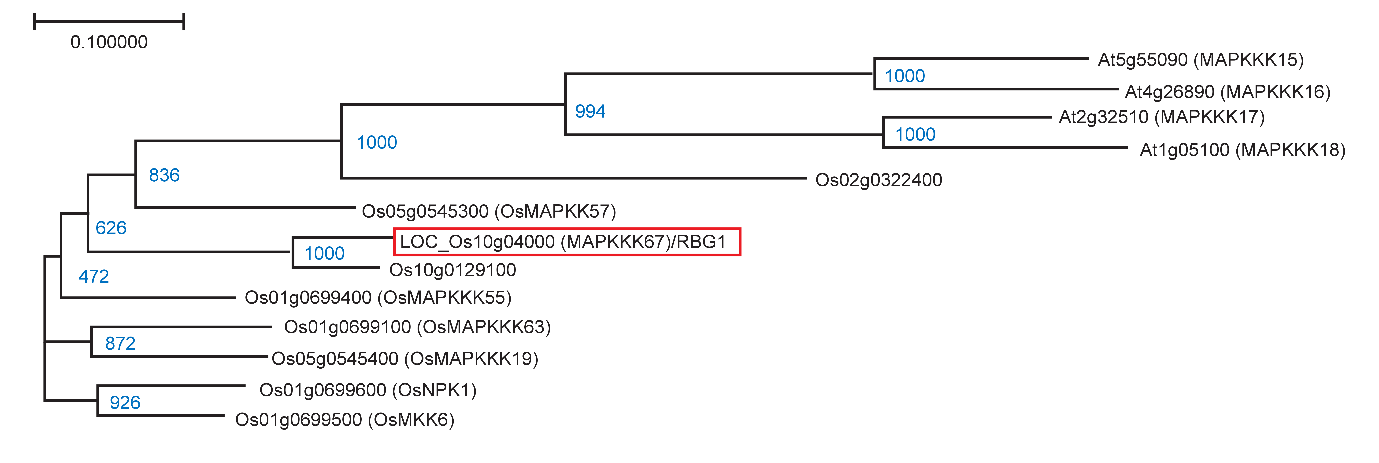


**Supplementary Figure S6.** **In vitro kinase assay of RBG1.** The full-size blots of Figure 3b. 1: OsMKK3 (50ng), 2: OsMKK3 (50ng) + GST-fused NB RBG1, 3: OsMKK3 (50ng) + GST-fused KO RBG1, 4: OsMKK3 (100ng), 5: OsMKK3 (100ng) + GST-fused NB RBG1, 6: OsMKK3 (100ng) + GST-fused KO RBG1, 7: OsMKK3 (150ng), 8: OsMKK3 (150ng) + GST-fused NB RBG1, 9: OsMKK3 (150ng) + GST-fused KO RBG1, 10: OsMKK3 (200ng), 11: OsMKK3 (200ng) + GST-fused NB RBG1, 12: OsMKK3 (200ng) + GST-fused KO RBG1. GST-RBG1 proteins (10 ng) were incubated with OsMKK3 in the kinase reaction mixture, and aliquots of the samples were separated by SDS-PAGE and subjected to autoradiography. The autoradiography images were obtained by 7-day exposure. The phosphorylation of OsMKK3 was observed by RBG1 proteins both of NB and KO type. Blots of GST-RBG1 proteins were detected by auto-phosphorylation. The protein molecular weight size marker is Bio-Rad Precision Plus Protein All Blue Standard (Bio-Rad).


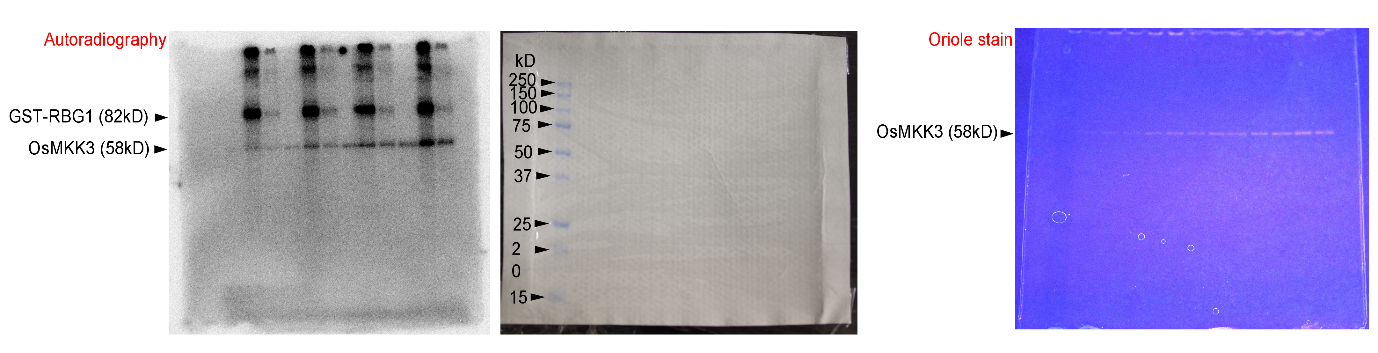


**Supplementary Figure S7. In vitro kinase assay of RBG1.** The full-size blots of Figure 3c. Lane 1 is OsMKK3 (substrate) only and Lane 2-8 are absent of OsMKK3. Lane 2 and 9: GST control, Lane 3 and 10: GST-fused NB RBG1, Lane 4 and 11: GST-fused KO RBG1, Lane 5 and 12: GST-fused RBG1 that has the A115T amino acid substitution (KO type) introduced into the NB type, Lane 6 and 13: GST-fused RBG1 that has the N130K amino acid substitution (KO type) introduced into the NB type, Lane 7 and 14: GST-fused RBG1 that has the G327C amino acid substitution (KO type) introduced into the NB type, Lane 8 and 15: GST-fused RBG1 that has the C524G amino acid substitution introduced into the NB type to eliminate kinase activity (negative control). GST-RBG1 proteins (10 ng) were incubated with OsMKK3 (200 ng) in the kinase reaction mixture, and aliquots of the samples were separated by SDS-PAGE and subjected to autoradiography. The autoradiography images were obtained by overnight exposure. Blots of GST-RBG1 proteins were detected by auto-phosphorylation. The protein molecular weight size marker is Bio-Rad Precision Plus Unstained Standard (Bio-Rad).


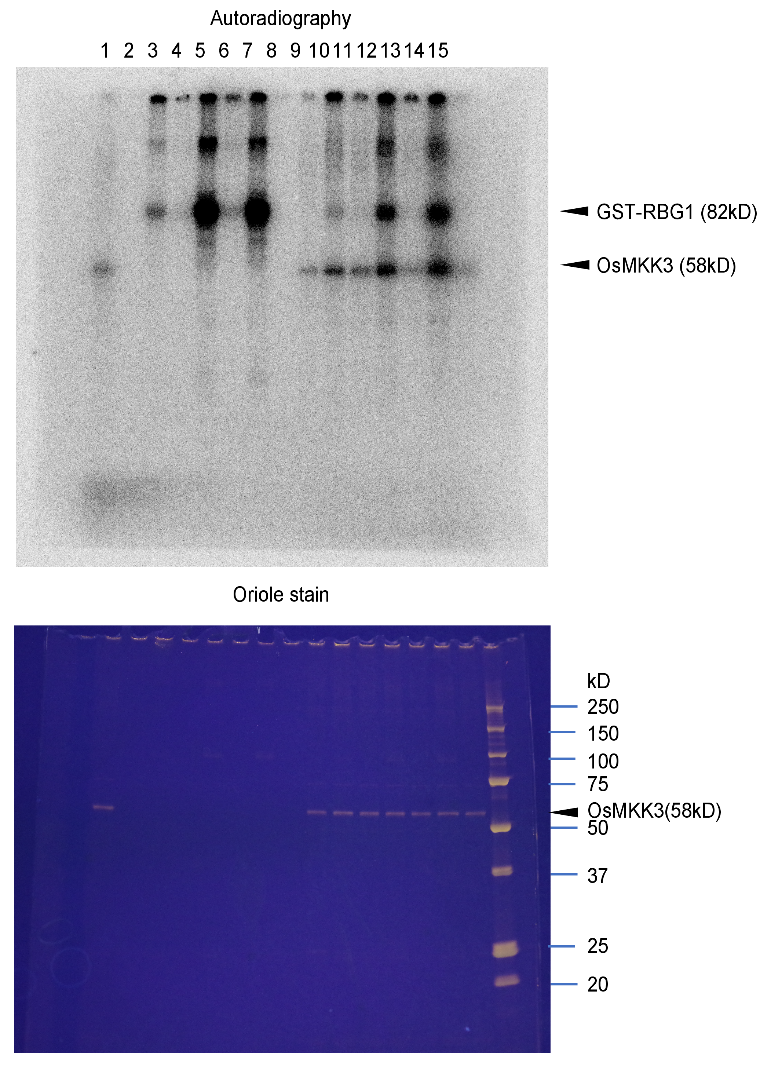


**Supplementary Figure S8. Comparison of tolerance to several abiotic stresses of ‘Koshihikari’ (KO) and RBG1res-NIL.** (a) Germination rates were measured under cold conditions (15 °C). Data are means ± s.d.; n=5 blocks (100 plants per block). There was no statistically significant difference between KO and RBG1res-NIL (Student’s *t*-test) for any treatment duration. (b) Germination rates were measured under hot conditions (37 °C). Data are means ± s.d.; n=5 blocks (100 plants per block). There was no statistically significant difference between KO and RBG1res-NIL (Student’s *t*-test) for any treatment duration. (c) Comparison of salt tolerance between ‘Koshihikari’ (KO), RBG1res-NIL, and ‘Nona Bokra’ (NB; salt tolerant) at the time of germination. Twenty seeds of each line were sown in soil and cultivated by adding water containing a high salt concentration (NaCl 0.375% or 0.75%), and photos were taken at 7 days after sowing. Scale bar represents 6 cm. (d) Drought treatment was applied to 14-day-old seedlings by withholding water for 6 days. After drought treatment, water was added to seedlings and cultivation was continued. Seedling survival was defined as the emergence of new leaves, and survival rate was measured at 7 days after the end of the drought treatment. Data are means ± s.d.; n=2 blocks (15 plants per block). There was no statistically significant difference between KO and RBG1res-NIL (Student’s *t*-test). (e) Typical images of KO and RBG1res-NIL at 7 days after the end of drought treatment. Scale bar represents 6 cm.


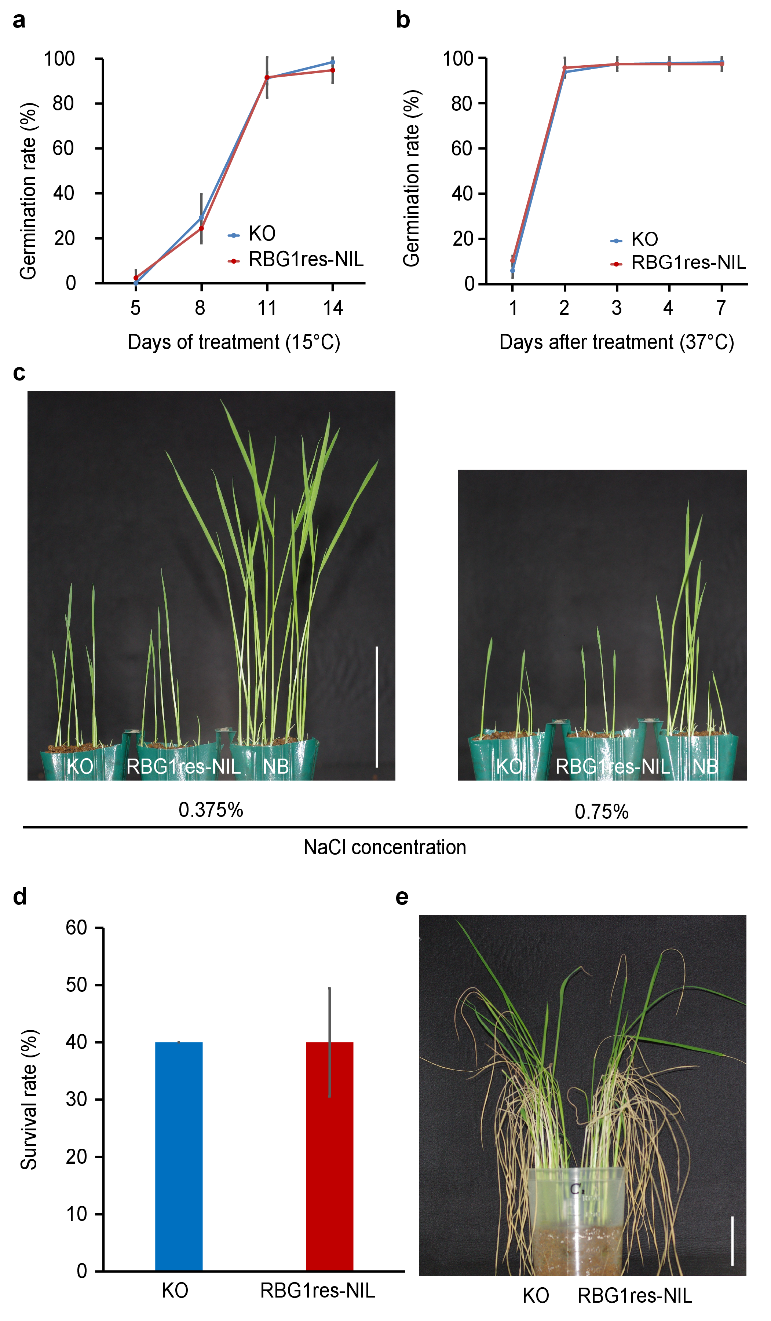


**Supplementary Figure S9. Expression analysis and germination characteristics of *RBG1*.** (a) *RBG1* expression in embryo and other tissues of KO and RBG1res-NIL. Embryo samples were taken from mature seeds 5 months after harvest. Shoot and root samples were taken 5 days after sowing, and young leaf and leaf blade samples were taken 14 and 51 days after sowing, respectively. Spikelet samples were taken 1 day after flowering. Expression data for *RBG1* were normalized to the expression of a ubiquitin gene. Data are means ± s.d.; n=3. There was no statistically significant difference between ‘Koshihikari’ (KO) and RBG1res-NIL (Student’s *t*-test) for any tissue. (b) *RBG1* expression in reproductive tissues of KO and RBG1res-NIL. Ovary samples were taken on the day of flowering, and embryo and endosperm were taken 7, 14, 28, and 42 days after flowering (DAF). Expression data for *RBG1* were normalized to the expression of a ubiquitin gene. Data are means ± s.d.; n=3. There was no statistically significant difference between KO and RBG1res-NIL (Student’s *t*-test) for any sample type. (c) Similar levels of seed dormancy of KO and RBG1res-NIL. Panicles were sampled 6 weeks after heading, wrapped with paper towels, and dipped in water. After the water was briefly drained off, the panicles were incubated in the dark at 30 °C. Photo was taken 7 days after treatment. Scale bar represents 5 cm. (d) Germination rates of KO and RBG1res-NIL at various time points (4 weeks (4W), 6 weeks (6W), 8 weeks (8W), and 10 weeks (10W)) after heading. Data are means ± s. d. (50 seeds from one panicle × 5 repeats). There was no statistically significant difference between KO and RBG1res-NIL (Student’s *t*-test) at any time point. (e) *RBG1* expression after inoculation of embryo and other tissues with *Burkholderia glumae* (*B. glumae*) in KO and RBG1res-NIL. Expression data for *RBG1* were normalized to the expression of a ubiquitin gene. Data are means ± s.d.; n=3. There was no statistically significant difference between KO and RBG1res-NIL (Student’s *t*-test) for any tissue–treatment combination.


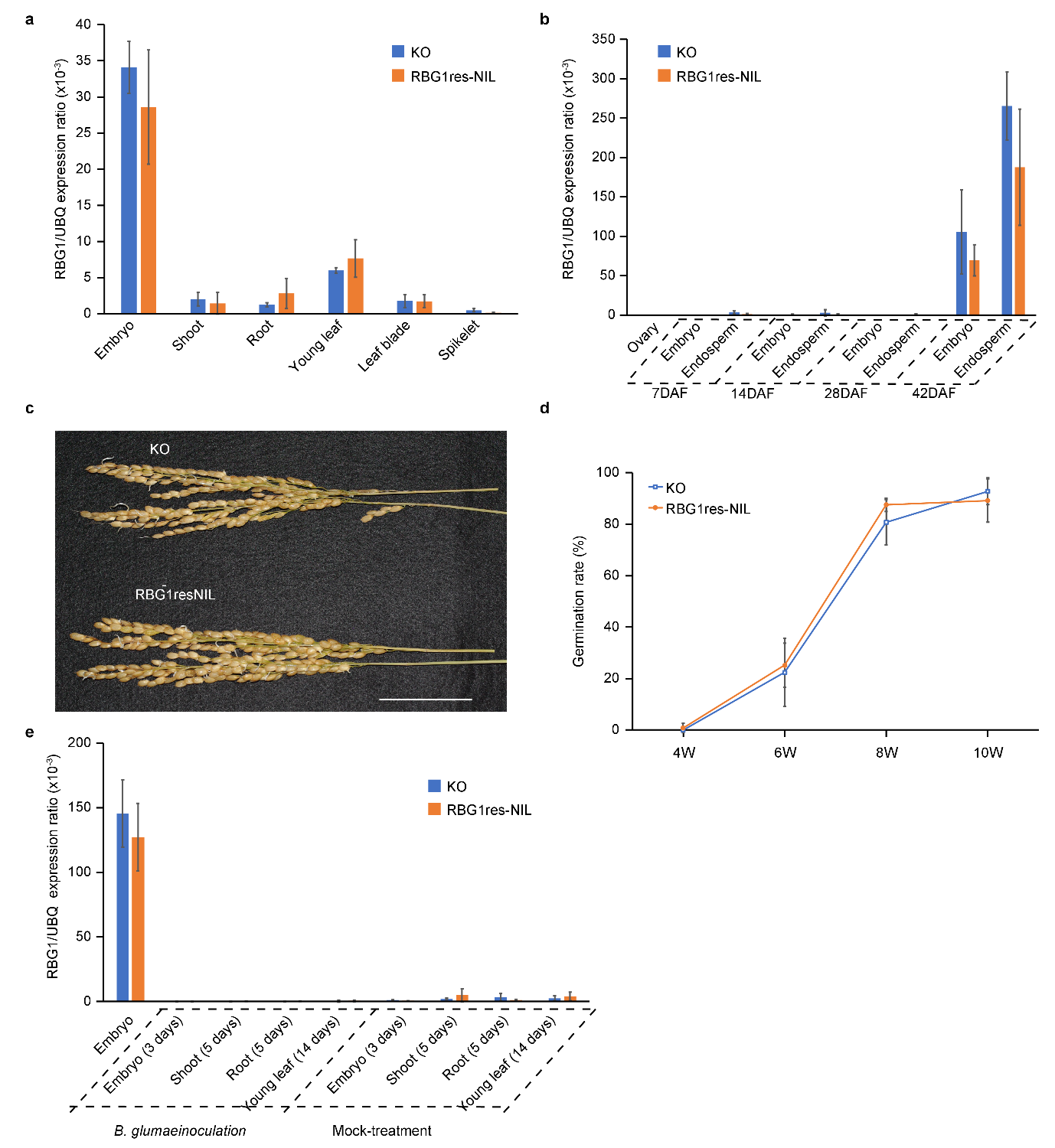


**Supplementary Figure S10.** **Expression of defense-related genes in embryos of ‘Koshihikari’ (KO) and RBG1res-NIL after inoculation with *B. glumae*.** Embryos 2 days after inoculation of mock (water) or *B. glumae* were analyzed. Expression data were normalized to the expression of a ubiquitin gene. Data are means ± s.d.; n=3. There was no statistically significant difference between KO and RBG1res-NIL (Student’s *t*-test) for any comparison.


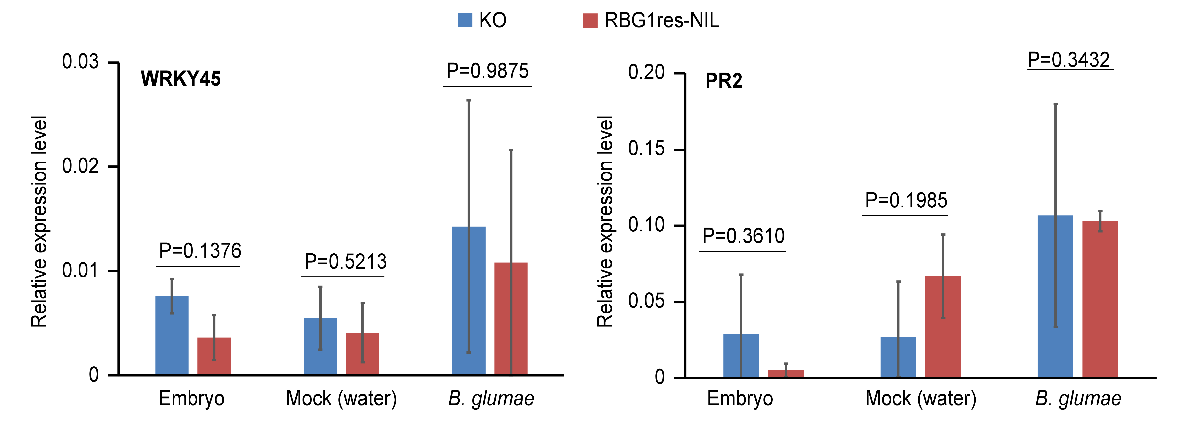


**Supplementary Figure S11. Reactions to several plant pathogens from vegetative to flowering stages of ‘Koshihikari’ (KO) and RBG1res-NIL.** (a, b) Resistance to bacterial grain rot caused by *Burkholderia glumae.* (a) Panicle disease scores were measured at 6 days after treatment by the cut-panicle inoculation method. Data are means ± s.d.; n=5. There was no statistically significant difference between KO and RBG1res-NIL (Student’s *t*-test). (b) Typical images of KO and RBG1res-NIL at 6 days after inoculation. Scale bar represents 6 cm. (c) Resistance to leaf blast caused by *Pyricularia oryzae.* Leaf blast disease scores were measured in a field test. Data are means ± s.d.; n=10. There was no statistically significant difference between KO and RBG1res-NIL (Student’s *t*-test). (d) Resistance to bacterial blight caused by *Xanthomonas* *oryzae* pv. *oryzae.* Flag leaves were inoculated by the clipping method. Lesion lengths were measured 20 days after infection with virulent *X. oryzae* pv. *oryzae* race 2 (T7147). Data are means ± s.d.; n=8. There was no statistically significant difference between KO and RBG1res-NIL (Student’s *t*-test).


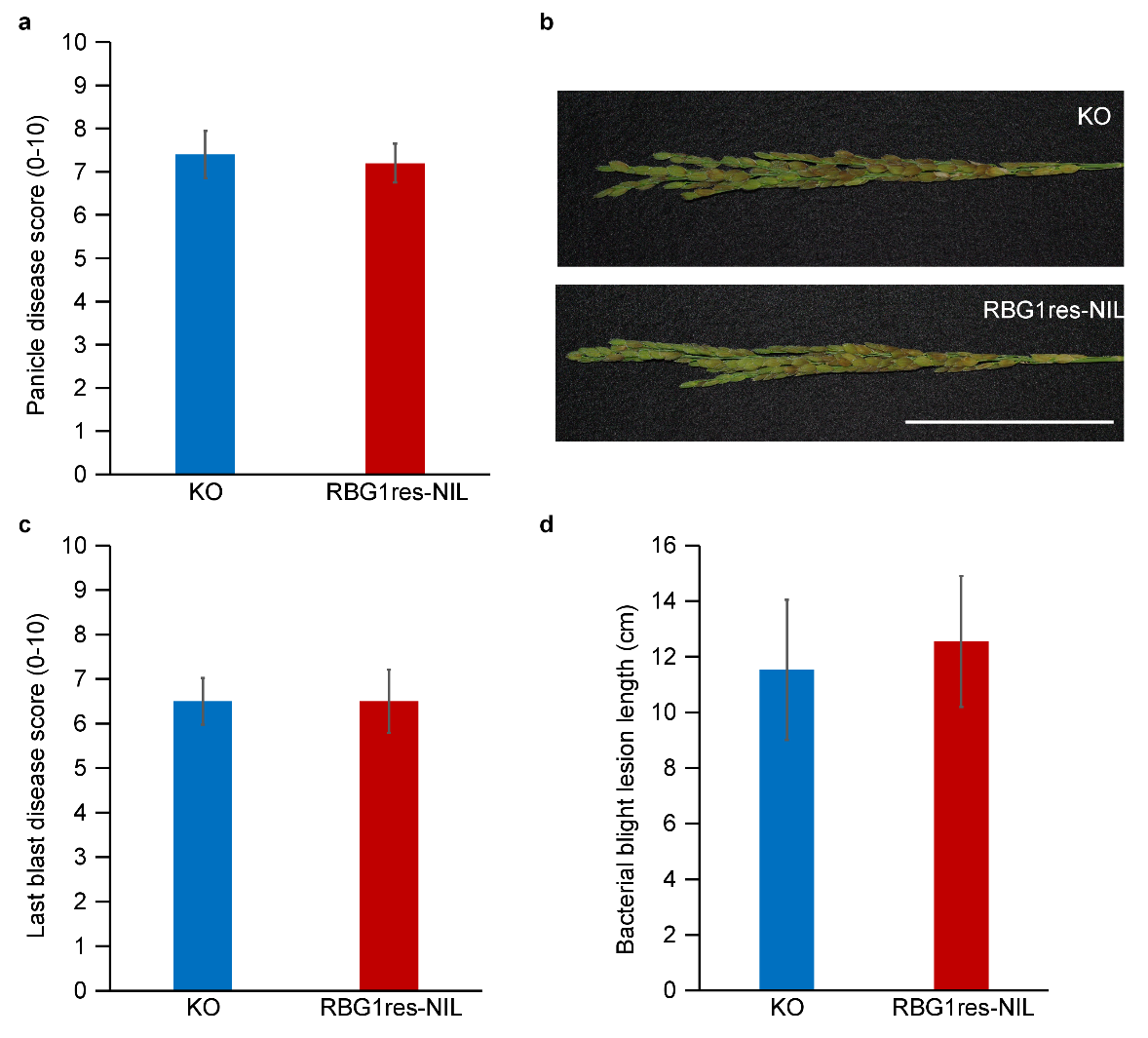

Supplement: Supplementary file 2 — Supplementary Figures. [file 41598_2023_30471_MOESM2_ESM.docx]
